# Cost-Effectiveness of the Chronic Disease Self-Management Program: Implications for Community-Based Organizations

**DOI:** 10.3389/fpubh.2015.00027

**Published:** 2015-04-27

**Authors:** Rashmita Basu, Marcia G. Ory, Samuel D. Towne, Matthew Lee Smith, Angela K. Hochhalter, SangNam Ahn

**Affiliations:** ^1^Baylor Scott & White Health, Temple, TX, USA; ^2^Department of Health Promotion and Community Health Sciences, School of Public Health, Texas A&M Health Science Center, College Station, TX, USA; ^3^Department of Health Promotion and Behavior, College of Public Health, The University of Georgia, Athens, GA, USA; ^4^Division of Health Systems Management and Policy, School of Public Health, The University of Memphis, Memphis, TN, USA

**Keywords:** chronic disease self-management, cost-effectiveness analysis, health-related quality of life, older adults, EQ-5D and quality-adjusted life years

## Abstract

Chronic conditions are the leading cause of growing healthcare spending, disability, and death in the U.S. In the wake of national health reform, policy makers and healthcare professionals are becoming increasingly concerned in containing healthcare costs while improving quality of patient care. A basic policy question is whether the Chronic Disease Self-Management Program (CDSMP), a widely distributed evidenced-based self-managed program, can be cost-effective in managing chronic conditions while improving quality of life. Utilizing data from the *National Study of CDSMP*, the primary objective of the current study is to estimate cost-effectiveness of the CDSMP program among individuals with at least one chronic condition. The second objective is to determine how cost-effectiveness ratios vary by depression status. EuroQol-5D (EQ-5D) was used to measure health-related quality of life (HRQOL) of CDSMP participants, which was then converted to quality-adjusted life years (QALYs) for cost-effectiveness analysis. Participants who completed the CDSMP program experienced higher EQ-5D scores from baseline to 12-month follow-up (increased from 0.736 to 0.755; *p* < 0.001). The incremental cost-effectiveness ratio (ICER) ranges from $83,285 to $31,285 per QALYs, which can be comparable to the common benchmark of $50,000/QALYs. ICER by baseline depression status indicates that it will cost more per QALYs gained for those diagnosed with depression based on their Patient Health Questionnaire-8 score. However, cautions should be taken while considering this point estimate too literally because the average cost for CDSMP participants was a rough estimate and based on several simplifying assumptions. Identifying cost-effective strategies that can lower the burden of chronic disease among community-dwelling adults is critical for decision makers in allocating limited resources. Policy makers and community organizations can use this information to guide funding decisions and delivery of CDSMP programs for individuals with multiple chronic health conditions.

## Introduction

With the rapid aging of the baby boomer cohort, it is estimated that one in five Americans will be 65 years or older by 2030 (1). Simultaneously, the existence of multiple chronic conditions among Americans 65 years or older is becoming increasingly prevalent, with 60–75% of older adults having at least two chronic conditions (2), many of which are preventable (1). Moreover, the number of Americans with chronic conditions is projected to increase by 37% by the year 2030 (3). More than 75% of total healthcare costs are attributable to the treatment of chronic illnesses (3). Furthermore, chronic conditions among older adults are associated with lower quality of life and increased limitations in activities of daily living (4–7).

With a mission to promote health and the quality of life in Americans, the U.S. Department of Health and Human Services’ Center for Disease Control and Prevention (CDC) has supported population surveillance of health-related quality of life (HRQOL) (8). HRQOL is a multi-dimensional measure, which is defined as “perceived physical and mental health over time” (9). It can be considered as a part of a person’s overall quality of life that is determined by his or her health status. Because HRQOL addresses physical and mental health of a large number of individuals, it can offer current health data that public health agencies need to assess population health. In light of the growing prevalence of chronic illness, healthcare burdens, and concerns for promoting population health, service providers and policy makers are pursuing cost-effective ways to design self-management programs that can improve the health and well-being of the population (10, 11). As healthcare costs continue to rise for treating chronic diseases, identifying ways to manage the progression of multiple chronic conditions among older adults is critical and time-sensitive (12, 13).

To promote health and the quality of life of community-dwelling older adults, federal, state, and local stakeholders are implementing evidence-based initiatives to engage individuals in managing chronic health conditions while improving health outcomes (14, 15). One such approach is the implementation of self-management programs that improve health and quality of life while simultaneously reducing costly healthcare utilization (16–20). These self-management programs have the potential to embrace the triple aim goals of healthcare (better care, improved patient care experience, and lower cost of care) that will enhance population health (21).

The Chronic Disease Self-Management Program (CDSMP), one of the well-studied evidence-based programs, improves health status and chronic illness symptoms while showing promise for lowering healthcare spending through the reduction in hospitalization (22, 23). Although evidence suggests that CDSMP can improve health outcomes among patients with chronic diseases (24–26), little is known about the *cost-effectiveness* of improving HRQOL among CDSMP participants. Moreover, program effectiveness may vary when chronic diseases are accompanied by depression because individuals with depression are less likely to complete the self-management education programs than those without depression (27). Thus, the current study has two goals: (1) to perform an economic evaluation of the CDSMP by utilizing a cost-effectiveness analysis of HRQOL among CDSMP participants from baseline to 6-month and 12-month follow-up; and (2) to examine how the intervention effectiveness varies for participants with or without depression at baseline.

## Materials and Methods

### National study of CDSMP as study basis

The current study utilized a change in HRQOL measures at three time points (baseline, at 6-month, and at 12-month) to examine the cost-effectiveness of the intervention among middle-aged and older adults enrolled in the *National Study of the Chronic Disease Self-Management Program (CDSMP)*. Data were analyzed from workshops delivered nationwide by 22 licensed sites in 17 states across the nation from August 2010 to April 2011. CDSMP workshops were supported by various federal, state and local sources, healthcare organizations, and community agencies. The eligibility criteria and recruitment, intervention delivery, and referral activities are described elsewhere (22). Sites already licensed to deliver CDSMP were selected and then agreed to participate in the *National Study*, delivering the manualized workshops following standardized intervention protocols and submitting data for study purposes. Data were collected in person before the start of the intervention (baseline) and at 6 and 12 months post-intervention by mail/phone. Investigators had no role in leader training, workshop recruitment, or program implementation. Each CDSMP delivery site recruited people for workshops in their usual fashion, which included referrals from organizations serving older adults (e.g., senior centers, healthcare facilities, and social service organizations as well as self-referrals from other recruitment activities including flyers, brochures, and health fairs). The intervention was designed to focus on content areas including (a) techniques to manage typical responses to chronic health problems such as frustration, fatigue, pain, and isolation: (b) improving healthy behavior such as physical exercise for maintaining and improving strength, flexibility, and endurance; and (c) appropriate use of medications, effective communication with healthcare professionals (24, 28). For the purpose of this study, participants with complete information on indicators of HRQOL at baseline, 6-month, and 12-month follow-up were included.

#### Study sample

As a part of translating this intervention, CDSMP included 1,170 community-dwelling individuals at baseline, 6 months, and 12 months across the nation. A total of 825 (71%) participants completed 12-month follow-up assessment including HRQOL measures and approximately 77% (*n* = 903) participants completed 6-month follow-up (29). While attrition was minimal for a community-based translational research study, HRQOL information at the 6-month follow-up data was missing for 77 participants (*N* = 748 contributed to the final analyses). Few differences were observed based on data attrition. Participants who completed follow-up assessments at 6-month and 12-month tended to be older, and completers of the 6-month assessment were more likely to be non-Hispanic White (15). Institutional Review Board approval for the *National Study* was obtained at Stanford University and Texas A&M University.

### Measures

#### Health-related quality of life measures

In the current study, we focus on the healthy-days measure of HRQOL because it captures the key concepts of population health and well-being. This construct is aligned with one of CDSMP’s main objectives of empowering program participants to better manage their chronic conditions and experience a higher quality of life. Healthy-days measures are important components that assess HRQOL. The HRQOL includes a set of four questions (8):

1. Would you say that in general your health is; Excellent, Very good, Good, Fair, or Poor?
2. Now thinking about your physical health, which includes physical illness and injury, for how many days during the past 30 days was your physical health not good?
3. Now thinking about your mental health, which includes stress, depression, and problems with emotions, for how many days during the past 30 days was your mental health not good?
4. During the past 30 days, for about how many days did poor physical or mental health keep you from doing your usual activities, such as self-care, work, or recreation?

In the current study, we utilize an “unhealthy days” summary measure, which is based on the second and third questions, estimates the overall number of days when physical and mental health was not good. We then calculate the number of days estimated to be healthy, which is the complement to unhealthy days measure (total number of “healthy days” limits to maximum of 30 days as this is the maximum possible value that this measure could possibly take). These items have been extensively used for evaluating program objectives in other studies (8, 30–33) and the validity of these measures has been confirmed in population based samples. Participants responded to each item with the number of days ranging from 0 to 30.

#### Cost measures

The average cost per CDSMP participant varies by the number of enrolled participants per workshop with the estimated per-participant cost ranging from $219 to $583 (23). In the *National Study of CDSMP*, 145 workshops had an average size of 12.7 (±4.18) participants, with the majority of workshops (66.2%) having between 8 and 16 participants. A detailed description of the cost measures reported by CDSMP delivery sites appears elsewhere (23). Based on extant literature confirmed by experts in the delivery field, we estimated program costs at $350 per participant, assuming an average of 10 participants in each CDSMP workshop (23). These program costs typically include licensure costs, trained peer personnel, materials, and any space rental costs (34). Because the CDSMP was a community-based program and goal that this type of self-management program is to provide evidence regarding resources needed to deliver within the community, cost data are collected at the aggregate level. In the case of CDSMP, per-participant costs were aggregated at the workshop level. Individual-level cost data are less valuable for the effective implementation of this type of community-based program.

#### Other participant-level measures

Participants’ demographic characteristics measured at baseline included age, gender, race/ethnicity, and number of chronic conditions. Measures and a sample questionnaire can be found in English and Spanish (35). Depressive symptoms were measured using the Patient Health Questionnaire (PHQ) (36). Self-rated items (9 DSM-IV criteria) scored from 0 (not at all) to 3 (nearly everyday) were added to determine overall PHQ score of study participants at baseline. A score greater than or equal to 10 was considered clinically depressed because this cut-off point of 10 has a sensitivity and specificity of 88% in detecting a diagnosis of major depression in primary care patients (36). The reliability of the PHQ-9 is high, with a Cronbach’s α of 0.89 and test–retest reliability of 0.84 (37). We used an eight-item version of the PHQ (38), which excludes the item that asks patients if they have been bothered by “thoughts that you would be better off dead or of hurting yourself in some way.” Scores for the eight-item version of the PHQ range from 0 to 24, and are highly correlated with scores on the nine-item version (*r* = 0.997) (38).

### Analysis

#### HRQOL, EQ-5D, and QALYs

The CDC-derived measure of HRQOL is one of the most commonly used outcome measures for evaluating burden of disease in public health research. However, a single measurement such as quality-adjusted life year (QALY) is considered as a more useful measure for cost-effectiveness analyses (38–40). This is because QALY uses preference-based measures of HRQOL, which uses summary scores (i.e., utility values) to represent population preferences for different health states. Because the number of unhealthy days are not preference-based measures of HRQOL (as asked in the CDSMP survey), the CDC “healthy days” measures cannot be used directly in the cost-effectiveness analyses (41). Since the CDSMP survey did not include preference-based measure of EQ-5D, the non-preference-based scores of “healthy days” measure was converted to preference-based EuroQol 5D (EQ-5D) utilizing the method proposed by Jia and Lubetkin (42). The EQ-5D is an internationally developed preference-based (29, 43) method that provides a measure of utility scores to calculate QALYs which is used in cost-effectiveness analyses (44). EQ-5D estimates are obtained from healthy days by matching the cumulative distributions of the two HRQOL measures and EQ-5D from Behavioral Risk Factor Surveillance System and Medical Expenditure Panel Survey datasets (42). For example, we obtained EQ-5D utility score corresponding to number of “healthy days” measures in our sample. Detail description of the estimation method including underlying assumptions can be found in Jia and Lubetkin (42). Utility values range from 1 (best possible health state) through 0 (death) (44). We then used EQ-5D scores to calculate QALY for the calculation of cost-effectiveness ratios.

Estimation of participant specific QALYs was based on partitioning the study period into the number of follow-up assessments and weighting each time interval by the individual’s utility score during that period of time (45). It is assumed that changes in utility values are linear over time, which is the most commonly used method in cost-effectiveness analysis. Individual-level QALYs are then estimated by applying the area-under the curve approach. Details of this method can be found elsewhere (42). The general expression for calculating QALYs using individual data that are fully observed (i.e., no censoring) can be written as follows (45):

(1) QALY=∑t=0nQ1+Q t+12×Tt+1+TtT

where, *n* is the number of utility measurements over the study period (i.e., 1 year), *Q*_t_ is the individual utility score (i.e., EQ-5D score) obtained in the *t*^th^ measurement, *T* is the total duration of study period expressed in terms of total number of time units in a year (e.g., months), *T*_t_ is the time period in which the *t*^th^ measurement takes place (expressed as number of time units in a year). In our case, *n* = 2 (i.e., first interval from baseline to 6-month and second interval from 6-month to 12-month), *T* = 12 (i.e., number of months in a year), and three time points as *T*_0_ = 0, *T*_1_ = 6, and *T*_2_ = 12. For example, in our current study, QALYs are obtained by substituting mean EQ-5D scores and controlling for baseline utility (45):

(2) $$\begin{array}{ll} & \mathrm{Incremental QALY}=\sum_{t=0}^{n}\left[\frac{\left(0.743-0.736\right)}{2}\times \frac{6}{12}+\frac{\left(0.743-0.736\right)}{2}\times \frac{6}{12}+\frac{\left(0.755-0.743\right)}{2}\times \frac{6}{12}\right]\\ & \;=0.007 \end{array}$$

#### Cost-effectiveness analysis

The strong need to control healthcare costs for the treatment of chronic diseases led us to search for interventions that produce greatest value, based on comparative economic evaluation (46). Cost-effectiveness analysis (CEA) is a type of economic evaluation method that can be utilized to assess whether money is well spent in a particular health promotion program (47). Funding agencies may continue to support programs on the basis of this information or may find additional interventions that can produce the best outcomes with available resources. The most widely used method for CEA is the incremental cost-effectiveness ratio (ICER), which compares differences in cost to differences in effectiveness between two competing interventions and therefore relevant for policy making decisions. In the absence of a control group, we are comparing health gains compared to no intervention (i.e., “doing nothing”). Therefore, in this case, ICER was calculated compared to baseline and measures the effectiveness of CDSMP in improving QALYs compared to “doing nothing.”

An important first step of calculating ICER is to quantify its average cost of a program in order to relate the cost to specific measures of the program (48). Considering zero cost for “no intervention,” the numerator of ICER is the average cost and denominator includes the mean effectiveness of the program (48, 49). In our case, the numerator is the mean program cost spent per CDSMP participant and denominator is QALYs estimates. The QALYs is particularly useful in quantifying program effectiveness and is the most commonly used measure of treatment effectiveness in CEA literature (50, 51). The ICER for each outcome measure was calculated by dividing per person CDSMP workshop costs by the QALYs. Therefore ICER can be indicated as:

(3) $$\mathrm{ICER=}\frac{\mathrm{Average cost spent per CDSMP participant}-\$0}{\mathrm{QALYs gained adjusted for baseline utility score}}$$

## Results

Table 1 describes participants’ characteristics at baseline. In total, 1,170 participants completed the baseline assessment. On average, participants were 65 years old, nearly 83% were female, and had an average of 13 years of education. Ethno-racial composition included 55% non-Hispanic white, 16% African American, 22% Hispanic, and 6.5% others. About 79% reported two or more conditions and 79.1% of participants attended four or more workshop sessions.

**Table 1:** **Sample characteristics at baseline (*N* = 1170)**.

Variables	% Mean (SD)
Age (in years)	65.4 (14.3)
Female	82.7
Race/ethnicity	–
Non-Hispanic White	55.2
African American	16.0
Hispanic	22.3
Other	6.5
Workshop completion rate	79.1
Education (1–23)	12.9 (3.8)
At least two chronic conditions	79.0
PHQ-8 depression (0–24)	6.6 (5.5)
Healthy days (0–30)	17.9 (11.5)
EQ-5D (0–1)^a^	0.736 (0.156)

*^a^In our sample, EQ-5D value ranges from 0.411 to 0.995*.

Table 2 represents summary statistics for healthy days and corresponding EQ-5D measures at baseline, 6-month, and 12-month during the study. Both healthy days (17.9–19.2) and corresponding EQ-5D scores (0.743–0.755) were significantly improved from baseline to 12-month) period (with a *p-*value <0.001); however, no significant improvement was observed for these measures from baseline to 6-month.

**Table 2:** **Changes in mean (SD) of healthy days and EQ-5D scores among CDSMP participants during the study period**.

HRQOL measures	Baseline	6 months	12 months	*p-*value for the change	
				Baseline and 6 months	Baseline and 12 months
Healthy days (0–30)	17.9 (11.5)	18.5 (11.4)	19.2 (11.1.)	0.25	<0.001
EQ-5D (0–1)	0.736 (0.156)	0.743 (0.156)	0.755 (0.152)	0.32	<0.001

Table 3 presents the similar statistics by baseline depression status. Changes in mean healthy days and EQ-5D scores were examined by utilizing paired *t*-test by baseline depression status. On average, participants with depression at baseline reported lower number of healthy days and their corresponding EQ-5D scores were also lower than participants who had no depression at baseline. However, both groups (depression versus no depression at baseline) showed significant improvement in healthy days and EQ-5D scores from baseline to the12-month period. These results indicate that CDSMP improves population health status among individuals with multiple chronic conditions through better disease self-management strategies.

**Table 3:** **Changes in mean (SD) of healthy days and EQ-5D scores by depression status at baseline**.

HRQOL measures	Depression at baseline (PHQ-8 ≥10)						Difference in scores: baseline and 6-months		Difference in scores: baseline and 12-months	
	Yes			No			Yes	No	Yes	No
	Baseline	6-months	12-months	Baseline	6-months	12-months		
Healthy days	16.4 (11.5)	17.3 (11.4)	17.9 (11.2)	24.1 (8.8)	24.9 (8.8)	27.2 (6.9)	0.33	0.52	0.01	0.001
EQ-5D	0.721 (0.15)	0.728 (0.15)	0.737 (0.15)	0.825 (0.11)	0.830 (0.13)	0.861 (0.10)	0.29	0.96	0.007	0.005

Table 4 shows the cost-effectiveness results for the CDSMP intervention. Incremental cost-effectiveness ratios (ICER) are calculated for the overall group as well as by baseline depression status. These ratios explain how much each additional QALYs gained with CDSMP will cost. Overall, ICER ranges from $83,285 to $31,285 per QALYs gained for participants in the CDSMP program with the median of $50,000/QALYs. ICER by baseline depression status indicates that it will cost more per QALYs gained for those diagnosed with depression based on PHQ-8 score.

**Table 4:** **ICER for QALYs for CDSMP participants**.

Overall						ICER by depression status based on PHQ-8 score											
QALYs = 0.007						Depressed at baseline QALYs = 0.006^a^						Not depressed at baseline QALYs = 0.026^b^					
Average cost-ranges			ICER = average cost/QALYs			Average cost-ranges			ICER = average cost/QALYs			Average cost-ranges			ICER = average cost/QALYs		
Max	Median	Min	Max	Median	Min	Max	Median	Min	Max	Median	Min	Max	Median	Min	Max	Median	Min
$583	$350	$219	$83,285	$50,000	$31,285	$583	$350	$219	$97166	$58333	$36500	$583	$350	$219	$22423	$13461	$8423

*^a^*${\mathrm{Incremental QALY}}_{\mathrm{depression}=\mathrm{yes}}=\left[\frac{\left(0.728-0.721\right)}{2}\times \frac{6}{12}+\frac{\left(0.728-0.721\right)}{2}\times \frac{6}{12}+\frac{\left(0.737-0.728\right)}{2}\times \frac{6}{12}\right]=0.006.$

*^b^*${\mathrm{Incremental QALY}}_{\mathrm{depression}=\mathrm{no}}=\left[\frac{\left(0.861-0.825\right)}{2}\times \frac{6}{12}+\frac{\left(0.861-0.825\right)}{2}\times \frac{6}{12}+\frac{\left(0.861-0.830\right)}{2}\times \frac{6}{12}\right]=0.026.$

## Discussion

Prior evidence suggests that CDSMP can significantly improve health outcomes for individuals with a variety of chronic conditions (21, 24). However, economic efficacy of the CDSMP on HRQOL is not well known. The current study developed a preference-based EQ-5D measure of HRQOL from healthy days to quantify the cost-effectiveness of a CDSMP program for improving QALYS gained for individuals with multiple chronic conditions. Although there is no universally acceptable threshold value for cost-effectiveness ratio (52), costs range from $50,000 to $75000 per QALYs gained have been considered as an acceptable value for resources expended (48).

Health-related quality of life is recognized as an important measure in public health as well as clinical research because it includes a population-based approach that addresses physical and mental health of a large number of individuals over time. Moreover, converting non-preference-based measures of HRQOL to a preference-based measure provides a way to compare the efficacy of CDSMP to other evidenced-based disease management programs in the literature. As shown in the current study, the economic value of CDSMP, as measured in dollars per QALYs gained, may have far reaching effects when magnified across the U.S. Thus, finding ways to improve the reach of the CDSMP among especially vulnerable individuals (e.g., rural, minority, low income) is a critical path of research for future studies.

Policy makers are interested in finding ways to improve the health of individuals with multiple chronic conditions as a significant share of healthcare dollars are attributed to the treatment of chronic medical conditions. Deploying resources with the goal of population-based health management will facilitate efficient allocation of resources in such a way that will lower overall healthcare cost, and improve quality of care experience (50). The CDSMP provides a mechanism to deliver cost-effective evidence-based strategies to those who may benefit most (e.g., being older and having co-morbid conditions). Poor quality of life and other mental health concerns has broader effects than immediate impacts on individuals with chronic conditions.

### Limitations

This study builds upon an existing translational *National Study of CDSMP*, which was not originally designed as an economic cost-effectiveness study. Hence, several variables typically included in economic analyses were not present (e.g., a comparison group or individual cost measures). While there was some participant attrition over time, the impacts appear minimal in terms of the diversity of participants in the study.

As an accommodation to available data, our study has assumed a standard per-participant costs now cited in the CDSMP literature (23). Although ICER values seem very attractive, cautions should be used to interpret too literally because these values can change substantially depending on changes in cost estimates assumption and point estimates. We acknowledge that this is a rough estimate that excludes a full consideration of all potential costs. One consideration is whether to include the opportunity cost of participating in the CDSMP program. Here, the opportunity cost would be the value of participation time in the workshop and which could be calculated based on wage forgone or the value of leisure time forgone. Since the majority of CDSMP participants are older adults, we can make the assumption that there is limited (if any) opportunity cost involved in terms of forgone wages as they are likely to be out of labor force. So, the value of leisure time could be the only way to calculate the opportunity cost of participating in the program. However, there is evidence that people do not always value the use of leisure time and it is also hard to estimate the value given the availability of survey instruments. The theoretical notion is that high motivation and retirement lower the time cost of participating in this type of health promotion program. Literature also suggests excluding time cost of participants in physical activity interventions (51).

Another limitation is that the calculation of QALYs was not adjusted for possible confounding factors which could potentially influence cots and outcome measures (45). Although the use of multiple regression method would control for this imbalance, the lack of a control group of CDSMP intervention makes this method infeasible in the current study. There are many more unmeasured benefits of the CDSMP that are not captured in the outcomes presented in the current study. For example, participants typically report many positive aspects of their participation including new social interactions. As such, the complete value of this evidence-based program is not fully measured in the current analysis and may be targeted for future study.

Using the generally accepted cost-effectiveness ratio of $50,000/QALYs, results of this study indicate that CDSMP is potentially cost-effective for individuals with multiple chronic conditions. Utilizing the most widely used generic measure of HRQOL from a population-based health days measure, the current study quantifies cost-effectiveness of CDSMP. However, due to the fact that CERs evaluate how a program’s costs compare to its outcomes, judgments about whether the outcomes achieved are worth than the cost are subjective and dependent on several factors (e.g., current needs and resources).

Nevertheless, we feel that this study makes a major contribution as one of the first studies to quantify the benefits of CDSMP in terms of a preference-based quality of life measure and examine the impacts for those with co-morbid depressive symptomatology. It provides a foundation for future cost-effectiveness studies of self-management programs for adults with multiple chronic conditions.

## Conflict of Interest Statement

Rashmita Basu has no conflicts of interest to disclose. Dr. Basu has no financial relationships with entities that could be perceived to influence, or that give the appearance of potentially influencing, what she wrote in the submitted work. Dr. Basu has no patents or copyrights to declare (whether pending, issued, licensed, and/or receiving royalties) relevant to the work. Dr. Basu has no other relationships or activities that readers could perceive to have influenced, or that give the appearance of potentially influencing, what she wrote in the submitted work. Marcia G. Ory has no conflicts of interest to disclose. Texas A&M University received a grant (PI: Dr. Ory) to evaluate the National Study of CDSMP. Dr. Ory has no financial relationships with entities that could be perceived to influence, or that give the appearance of potentially influencing, what she wrote in the submitted work. Dr. Ory has no patents or copyrights to declare (whether pending, issued, licensed, and/or receiving royalties) relevant to the work. Dr. Ory has no other relationships or activities that readers could perceive to have influenced, or that give the appearance of potentially influencing, what she wrote in the submitted work. Samuel D. Towne Jr. has no conflicts of interest to disclose. Neither myself nor this institution at any time received payment or services from a third party for any aspect of the submitted work. Dr. Towne has no financial relationships with entities that could be perceived to influence, or that give the appearance of potentially influencing, what he wrote in the submitted work. Dr. Towne has no patents or copyrights to declare (whether pending, issued, licensed, and/or receiving royalties) relevant to the work. Dr. Towne has no other relationships or activities that readers could perceive to have influenced, or that give the appearance of potentially influencing, what he wrote in the submitted work. Matthew Lee Smith has no conflicts of interest to disclose. Neither myself nor this institution at any time received payment or services from a third party for any aspect of the submitted work. Dr. Smith has no financial relationships with entities that could be perceived to influence, or that give the appearance of potentially influencing, what he wrote in the submitted work. Dr. Smith has no patents or copyrights to declare (whether pending, issued, licensed, and/or receiving royalties) relevant to the work. Dr. Smith has no other relationships or activities that readers could perceive to have influenced, or that give the appearance of potentially influencing, what he wrote in the submitted work. SangNam Ahn has no conflicts of interest to disclose.

*This paper is included in the Research Topic, “Evidence-Based Programming for Older Adults.” This Research Topic received partial funding from multiple government and private organizations/agencies; however, the views, findings, and conclusions in these articles are those of the authors and do not necessarily represent the official position of these organizations/agencies. All papers published in the Research Topic received peer review from members of the Frontiers in Public Health (Public Health Education and Promotion section) panel of Review Editors. Because this Research Topic represents work closely associated with a nationwide evidence-based movement in the US, many of the authors and/or Review Editors may have worked together previously in some fashion. Review Editors were purposively selected based on their expertise with evaluation and/or evidence-based programming for older adults. Review Editors were independent of named authors on any given article published in this volume*.
